# Prevalence, diagnosis, and clinical significance of lumbosacral transitional vertebrae: A systematic review and narrative analysis

**DOI:** 10.1016/j.bas.2025.105628

**Published:** 2025-10-07

**Authors:** Hanmo Fang, Jiayu Wang, Jackson Cosmas Kiwale, Min Cui, Xianlin Zeng, Cao Yang, Yukun Zhang, Lin Xie

**Affiliations:** a Department of Orthopaedics, Union Hospital, Tongji Medical College, Huazhong University of Science and Technology, 430022, Wuhan, China; b Department of Medical Ultrasound, Tongji Hospital, Tongji Medical College, Huazhong University of Science and Technology, Wuhan, 430030, Hubei Province, China

**Keywords:** Lumbosacral transitional vertebrae, Lumbar vertebra, Sacral vertebrae, Low back pain, Far-out syndrome, Systematic review

## Abstract

**Introduction:**

Lumbosacral Transitional Vertebrae (LSTV) are congenital anomalies at the L5-S1 segment, involving sacralization of the lowest lumbar vertebra or lumbarization of the highest sacral segment. Despite their clinical importance, a comprehensive systematic review addressing the prevalence, diagnosis, and clinical significance of LSTV remains limited.

**Research question:**

This study aims to systematically review and synthesize current evidence regarding the prevalence, diagnostic methods, and clinical significance of LSTV.

**Material and methods:**

An extensive literature search was undertaken across PubMed, Embase, and Medline databases to identify studies pertinent to the prevalence, diagnostic criteria, and clinical outcomes related to LSTV. All studies that satisfied our inclusion and exclusion criteria were methodically analyzed and included in this systematic review.

**Results:**

The mean prevalence of LSTV is 15.3 %, ranging from 4.5 % to 35.6 %, with higher occurrence in patients presenting with low back pain, disc herniation, and Adolescent Idiopathic Scoliosis (AIS). LSTV is diagnosed through imaging techniques such as X-ray, CT, and MRI, while symptomatic LSTV and Far-Out Syndrome (FOS) require clinical assessment, imaging, and diagnostic injections. LSTV is associated with an increased risk of nerve root compression, disc herniation, altered spinal pelvic parameters, and surgical complications including screw misplacement and vascular injury.

**Discussion and conclusion:**

Given LSTV's prevalence, surgical planning, including segment election and precise measurements, must account for LSTV's presence and its impact on disease progression. More research with larger samples and longer follow-ups is needed to elucidate these clinical implications and enhance therapeutic strategies.

## Highlights

• LSTV is closely linked to low back pain and disc herniation.
• LSTV complicates surgery due to anatomical and vascular changes.
• LSTV affects fusion level choice and outcomes in AIS patients.

## Abbreviations

AIS: Adolescent Idiopathic Scoliosis
ALIF: Anterior Lumbar Interbody Fusion
ASD: adjacent segment disease
FOS: Far-Out Syndrome
GRADE: Grading of Recommendations Assessment, Development, and Evaluation
LBP: low back pain
LSTV: Lumbosacral Transitional Vertebrae
OLIF: Oblique Lateral Interbody Fusion
PELD: Percutaneous Endoscopic Lumbar Discectomy
SD: standard deviation

## 1 Introduction

Lumbosacral Transitional Vertebrae (LSTV) is a prevalent anatomical anomaly found in the lumbosacral spine region. They are characterized by the elongation of transverse processes in the terminal vertebra, leading to either unilateral or bilateral articulations or osseous fusion with the sacral ala (Hughes & Saifuddin, 2004). LSTV manifests in two primary forms: the sacralization of lumbar vertebrae and the lumbarization of sacral vertebrae (Mahato, 2013a). Specifically, when the L5 vertebra is entirely fused with the sacrum, the spine consists of four lumbar vertebrae. Conversely, the detachment of the S1 vertebra from the sacrum results in six lumbar vertebrae (Paik et al., 2013). Additionally, intermediate forms, known as incomplete transitional vertebrae, are also categorized under LSTV (Paik et al., 2013; Mahato, 2013b). LSTV can originate from either the L5 or S1 vertebrae, displaying characteristics of both lumbar and sacral vertebrae. An LSTV that originates from L5 but resembles S1 is termed a “sacralized L5 vertebra”. In contrast, an LSTV originating from S1 and resembling an L5 vertebra, particularly when a distinct lower intervertebral gap is present, is defined as a “lumbarized S1 vertebra".

**Fig. 1:**
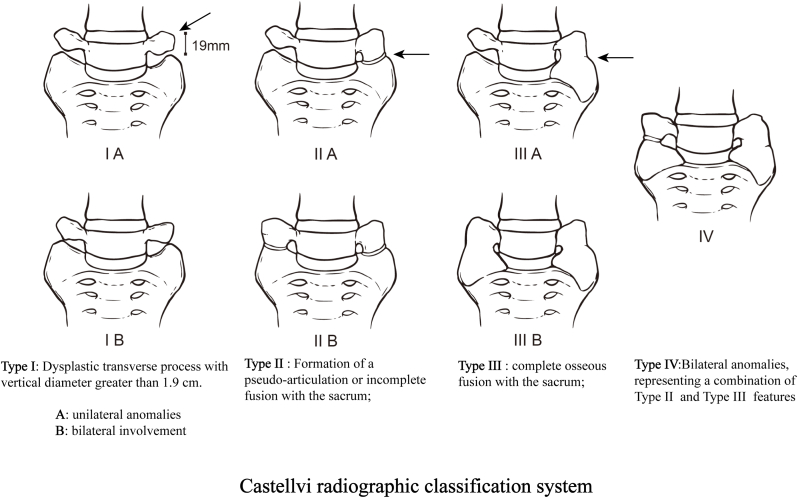
Castellvi radiographic classification system.

In 1984, Castellvi introduced a classification for LSTV based on the sacrum's coronal morphology (Fig. 1), delineating four types: Type I features dysplastic transverse processes (vertical diameter >1.9 cm); Type II involves the formation of a pseudo-articulation or incomplete fusion with the sacrum; Type III is characterized by complete osseous fusion with the sacrum; and Type IV represents a combination of Types II and III (bilateral anomalies). Unilateral anomalies in Types I, II, and III are marked as a, while bilateral involvement is indicated as b (Castellvi et al., 1984).

LSTV has garnered significant attention since 1917, primarily due to suspicions of its role in low back pain (LBP) (Bertolotti, 1917). Previous studies have highlighted various clinical implications of LSTV, including degenerative changes above the LSTV, degenerative alterations in the articulation between LSTV and the sacrum, facet joint arthropathy opposite unilateral fusion or pseudo-articulation LSTV, and foraminal stenosis due to underdeveloped transverse processes (Jancuska et al., 2015). Nevertheless, the clinical significance of these findings continues to be a subject of debate.

This systematic review seeks to address three critical questions: 1. What is the prevalence of LSTV? 2. What diagnostic methods are utilized for LSTV? 3. What clinical significance does LSTV hold?

## 2 Materials and methods

This study was approved by the Ethics Committee of Wuhan Union Hospital. Since all data were sourced from studies already in the public domain, the requirement for informed consent was bypassed. This systematic review was conducted strictly in accordance with the Preferred Reporting Items for Systematic Reviews and Meta-Analyses (PRISMA) guidelines (Moher et al., 2009).

### 2.1 Literature search and selection

On December 28, 2023, we embarked on a thorough search across the PubMed, Embase, and Cochrane databases, employing a blend of keywords: “LSTV,” “sacralization of lumbar vertebra,” and “lumbarization of sacral vertebra,” without imposing any additional constraints.

Inclusion criteria were (Hughes & Saifuddin, 2004): prospective or retrospective studies (Mahato, 2013a); studies on the prevalence, diagnosis, or clinical impact of LSTV. The following studies were excluded from this systematic review (Hughes & Saifuddin, 2004): cellular or animal experiments (Mahato, 2013a); case reports or reviews (Paik et al., 2013); studies with duplicate patient data (Mahato, 2013b); studies lacking valid data or full text.

## 3 Data collection

From the studies we meticulously extracted data encompassing author credentials, study methodology, geographical location, participant count, patient gender and average age, alongside specifics concerning LSTV such as its prevalence, diagnostic approaches, and clinical relevance.

## 4 Level of evidence

The quality of included studies was independently assessed by two authors using the Grading of Recommendations Assessment, Development, and Evaluation (GRADE) system (Guyatt et al., 2008). According to GRADE guidelines, each included study was rated as high, moderate, low, or very low quality based on scoring in five domains: risk of bias, inconsistency, indirectness, imprecision, and other considerations. The Kappa coefficient was calculated to determine the inter-rater reliability of the authors' ratings.

## 5 Statistical analysis

This systematic review's statistical evaluations were performed using SPSS software, version 22.0. Continuous variables were articulated as “mean ± standard deviation (SD)," while categorical variables were denoted as “number/percentage.” The notable heterogeneity and relatively small sample sizes of the studies under review led to the decision against performing a meta-analysis; a narrative synthesis was opted for instead.

## 6 Literature search and quality assessment

**Fig. 2:**
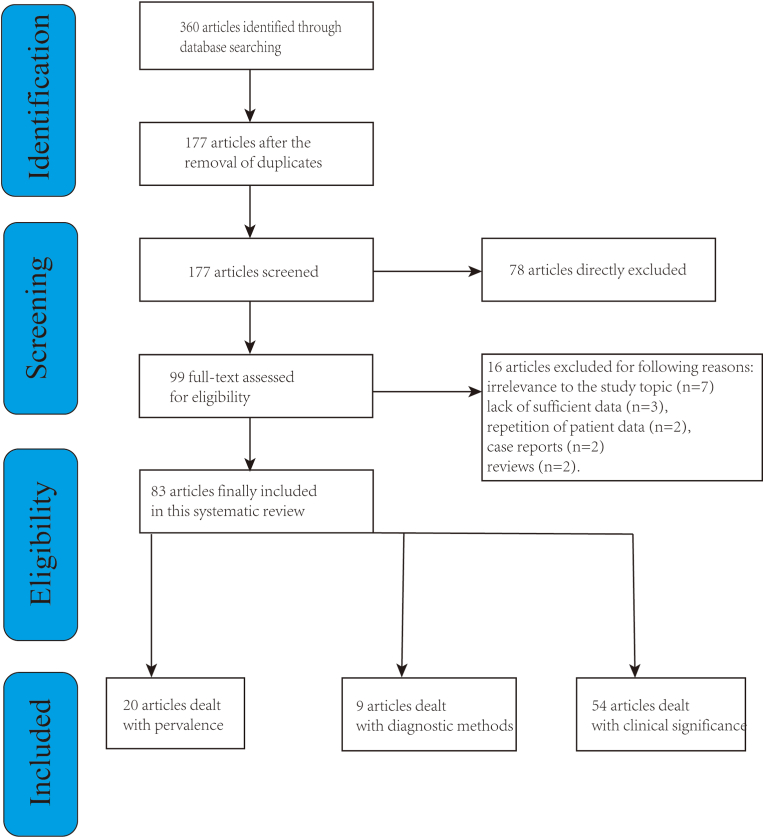
A flow chart of study selections.

We searched 360studies from the PubMed, Embase, and Cochrane databases. Upon the elimination of duplicate entries, 177 studies were subjected to further scrutiny. Of these, 78 were promptly excluded based on an initial review of their titles and abstracts. The remaining 99 underwent a detailed full-text evaluation, resulting in 16 being discarded due to reasons such as irrelevance to the study topic (n = 7), lack of sufficient data (n = 3), repetition of patient data (n = 2), being case reports (n = 2), or reviews (n = 2). This process culminated in the inclusion of 83 studies within this systematic review (Fig. 2). Employing the GRADE methodology, the quality of the included studies was systematically assessed, revealing that all were of either low or very low quality.

## 7 Results

### 7.1 Epidemiology and prevalence

20 studies have reported on the prevalence of LSTV (Table 1) (Chang & Nakagawa, 2004; Elster, 1989; Taskaynatan et al., 2005; Peh et al., 1999; Seçer et al., 2009; Sekharappa et al., 2014; Apaydin et al., 2019; Luoma et al., 2004; French et al., 2014; Otani et al., 2001; Hanhivaara et al., 2020; Nardo et al., 2012; Vinha et al., 2022; Delport et al., 2006; Apazidis et al., 2011; Tini et al., 1977; Wigh & Anthony, 1981; O'Driscoll et al., 1996; Chiu et al., 2024; Hahn et al., 1992). Geographically, 8 studies were conducted in Europe, 6 in the USA, 5 in Asia, and 1 in Australia. The prevalence of LSTV demonstrates variability across different populations, showing a higher incidence in males than in females, and is significantly elevated among individuals suffering from LBP, lumbar disc herniation, and AIS. On average, the prevalence of LSTV stands at approximately 15.3 % (4290 out of 28106), with a range from 4.5 % to 35.6 %.

**Table 1:** The prevalence of LSTV.

Study	Country	Study design	Population	Number of patients	Prevalence of LSTV (%)
Tini et al. (1977)	Switzerland	Retrospective	LBP Normal Population	269/4000 94/1873	6.7 % 5 %
Wigh and Anthony (1981)	USA	Retrospective	Herniation	42/200	21 %
Elster (1989)	USA	Retrospective	LBP	140/2000	7 %
Hahn et al. (1992)	USA	Retrospective	LBP	24/200	12 %
O'Driscoll et al. (1996)	UK	Retrospective	LBP	15/100	15 %
Peh et al. (1999)	China	Retrospective	LBP	17/141	13.2 %
Otani et al. (2001)	Japan	Retrospective	LBP Asia population normal population	64/501 55/508	13 % 11 %
Chang & Nakagawa, 2004	Japan	Retrospective	Herniation	10/62	16 %
Luoma et al. (2004)	Sweden	Retrospective	LBP	2,4/25 30,21/164	8 % vs 16 % 18 % vs 13 %
Taskaynatan et al. (2005)	Turkey	Retrospective	LBP	48/881	5.4 %
Marley et al. (2006)	USA	Retrospective	LBP	90/300	30 %
Seçer et al. (2009)	Turkey	Retrospective	LBP	18/62	4.5 %
Apazidis et al. (2011)	USA	Retrospective	American general population	75/211	35.6 %
Nardo et al. (2012)	USA	Retrospective	Osteoarthritis Initiative	841/4636	18.1 %
French et al. (2014)	Australia	Retrospective	Australian Population	588/5941	9.9 %
Sekharappa et al. (2014)	India	Retrospective	Urology outpatients Spine outpatients Discectomy patients	81/1000 140/1000 169/1000	8.1 % 14 % 16.9 %
Apaydin et al. (2019)	Turkey	Retrospective	LBP	600/1875	32 %
Hanhivaara et al. (2020)	Finland	Retrospective	Caucasian population	1101/3855	29 %
Vinha et al. (2022)	Portugal	Retrospective	Southern European population	142/571	24.9 %
Chiu et al. (2024)	Malaysia	Retrospective	AIS	250/998	25.1 %

### 7.2 Diagnostic methods

9 studies have detailed the diagnostic approaches for LSTV (Paik et al., 2013; Castellvi et al., 1984; Bertolotti, 1917; Peh et al., 1999; Wigh & Anthony, 1981; Chiu et al., 2024; Hahn et al., 1992; McGrath et al., 2021a; Nicholson et al., 1988). Bertolotti first highlighted the link between LSTV and LBP in 1917 (Bertolotti, 1917), introducing Bertolotti's syndrome. While LSTVs are detectable via X-rays, they are ideally visualized using Ferguson's view (an anteroposterior X-ray angled at 30° cranially). In 1981, Wigh and Anthony delineated the “squaring” presentation of transitional vertebrae on lateral views with a ratio of 1.37 between the anterior and posterior heights of the vertebrae's endplates (Wigh & Anthony, 1981). This “squaring” and “wedging” illustrate vertebral morphological changes, thereby deeming it an unreliable indicator for LSTV identification. In 1984, Castellvi and colleagues introduced a radiological classification for LSTV based on the transverse processes' morphological traits, categorizing LSTV into four distinct types (Castellvi et al., 1984). Nicholson and others in 1988 discussed the underdevelopment and reduced height of the intervertebral disc between L5 and S1 in cases of sacralization (Nicholson et al., 1988). Moreover, lumbarization instances showed that the intervertebral gap between S1 and S2 exceeds the residual disc space in the absence of LSTV. O'Driscoll and team later employed sagittal MR imaging to devise a four-type classification for the S1∼2 disc morphology, dependent on the disc material's presence and the anteroposterior diameter (O'Driscoll et al., 1996). In 2013, Paik emphasized the critical nature of accurate vertebral segment numbering, warning that errors could lead to surgical missteps (Paik et al., 2013). Notably, standard numbering from C2, assuming seven cervical and twelve thoracic vertebrae, does not account for variations like thoracic lumbarization or lumbar thoracization, requiring careful evaluation with anteroposterior spinal X-rays. In essence, X-rays and CT scans offer means to diagnose transverse process morphology, and sagittal imaging provides insights into the terminal vertebra and the underlying disc's shape, with comprehensive spinal imaging enhancing diagnostic precision. Nonetheless, the diagnostic reliability of these methodologies warrants further investigation due to the limited sample size of the existing studies.

### 7.3 The clinical impact of LSTV

A total of 26 studies were included that discussed the impact of LSTV on various conditions (Paik et al., 2013; Castellvi et al., 1984; Bertolotti, 1917; Elster, 1989; Otani et al., 2001; Vinha et al., 2022; Kikuchi et al., 2013; Yao et al., 2019; Avimadje et al., 1999; Vergauwen et al., 1997; Stein et al., 2023; Heo et al., 2019; Kanematsu et al., 2020; McGrath et al., 2021a,b; Cheng et al., 2022; Zhang et al., 2017; Becker et al., 2022; Byvaltsev et al., 2023; Jenkins et al., 2023a; Yamauchi et al., 2023; Jin et al., 2021; Lee et al., 2015; Illeez et al., 2022; Ichihara et al., 2004; Kapetanakis et al., 2025; Shen et al., 2023). The anatomical anomalies associated with LSTV lead to an increased proportion of diseases such as low back pain, and we systematically reviewed literature on the correlation between LSTV and disease. 12 studies described the association between LSTV and LBP, while 14 studies addressed the relationship between LSTV and degeneration of the intervertebral discs in the segments above.

LSTV is related to other anatomical and biomechanical changes, posing additional surgical risks to patients. Our goal was to systematically review and describe the surgical risks associated with LSTV, with 26 studies reporting significant associations with surgical risks (Mahato, 2011, 2013a; Chang & Nakagawa, 2004; Jenkins et al., 2023a, 2023b; Lian et al., 2018; Hou et al., 2018; Suzuki, 1987; Becker et al., 2023; Lisheng et al., 2023; Iwasaki et al., 2017; Zileli et al., 2024; Huang et al., 2021; Takata et al., 2014; Li et al., 2014; Moreau et al., 2016; Shin et al., 2019; Santavirta et al., 1993; Gong et al., 2023; Ahn et al., 2017; Ono et al., 2018; Paterson, 1893, 1894; Almeida et al., 2009; McCulloch & Waddell, 1980; Chung et al., 2021; Weiner et al., 2001). 16 studies discussed the considerations of segmental errors in patients with LSTV, 2 studies described the differences in pedicle screw placement due to LSTV, 4 studies explored the impact of LSTV on the clinical efficacy and recurrence rates of discectomy surgeries, and 5 studies indicated that the increased difficulty of anterior approach surgeries in patients with LSTV could lead to catastrophic vascular injuries.

## 8 Discussion

### 8.1 The association between lumbosacral transitional vertebrae and low back and leg pain: Bertolotti's syndrome and Far-Out Syndrome (FOS)

**Fig. 3:**
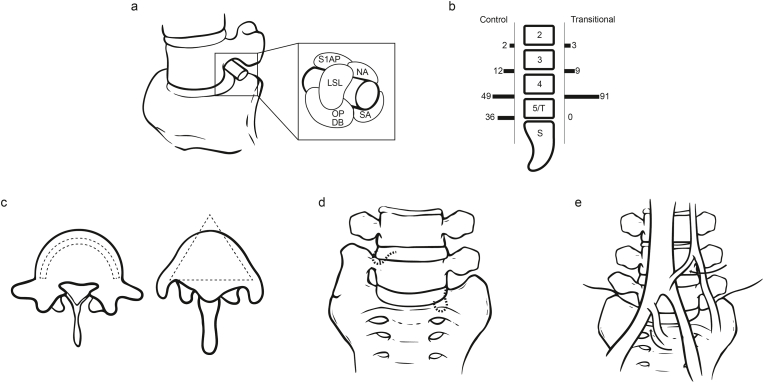
The Clinical Significance of LSTV. a. LSTV leads to nerve root compression, identified as FOS (Kanematsu et al., 2020). b. Disc herniations were more likely to occur at the segment above LSTV (Elster, 1989). c. Sacral lumbarization (semi-circular shape) and lumbar sacralization (triangular shape) (Mahato, 2011). d. The heightened risk of re-herniation associated with LSTV (Shin et al., 2019). e. The median sacral and ascending lumbar vessels may exhibit enlargement in LSTV (Weiner et al., 2001).

In a 2013 study by Paik et al., involving 8280 patients with LBP, Types II, III, and IV LSTV were found in 10.6 % of cases, with sacralization and lumbarization each accounting for 5.3 % (Paik et al., 2013). The relationship between LSTV and LBP was initially highlighted by Bertolotti in 1917, coining it as “Bertolotti's Syndrome” (Bertolotti, 1917). It is believed by some researchers that Types II and IV of LSTV are linked to LBP. The formation of a pseudo-articulation between the transverse process and the sacrum, susceptible to arthritic changes and osteophyte formation, may lead to nerve root compression, identified as FOS (Fig. 3a). The presence of LSTV can result in the lumbosacral nerve being pinched between the transverse process of the L5 vertebra and the sacral ala, termed “FOS Syndrome.” Osteoporosis due to “micromotion” at an underdeveloped facet joint below the transitional vertebra can cause extraforaminal entrapment of the spinal nerve, leading to radiculopathy. In a study of neural compression caused by new bone formation below LSTV, a 13 % incidence rate was observed, with up to 70 % of those cases being symptomatic (Porter et al., 2014).

Quinlan et al. reported a 4.6 % prevalence of Bertolotti's Syndrome in the general population and 11.4 % among patients under the age of 30 (Quinlan et al., 2006). Nonetheless, the potential connection between LSTV and LBP remains a topic of debate. For example, Nardo et al., in a cohort of 4636 osteoarthritis patients, noted that 53.9 % without LSTV reported LBP. Interestingly, only 46 % of Type I and 40 % of Type III LSTV patients reported LBP, suggesting these types might serve as protective factors. In contrast, 73 % of Type II and 66 % of Type IV LSTV patients reported LBP, highlighting a notable association with LBP (Nardo et al., 2012). On the other hand, some researchers argue that LSTV is relatively common in the general population and does not exhibit a higher prevalence among patients with LBP complaints. In 1977, Tini et al. identified no significant difference in LSTV prevalence between LBP sufferers (6.7 %, n = 4000) and the general populace (5 %, n = 1873) (Tini et al., 1977). The conflicting conclusions mentioned above may stem from the more comprehensive examinations conducted on the patient group in Tini's paper, whereas only simplified assessments were performed on the control group. This oversight could have led to undiagnosed cases within the control group, thereby inflating the prevalence rate of the control group. In recent studies, it has been found that accessory ossicles in the lumbosacral and hip regions may also cause nerve compression and limited spinal mobility. Additionally, the incidence of these ossicles varies significantly across different populations, which may contribute to heterogeneity in research findings (Ogut, 2025a, 2025b).

### 8.2 LSTV and Its Association with Degenerative Lumbar Conditions

Pain in the presence of LSTV may also be from lumbar degeneration, such as disc herniation, facet joint degeneration, spinal stenosis, or foraminal stenosis. A 1989 radiological analysis by Elster and colleagues on 2000 patients showed that structural pathologies like disc herniation, neural foraminal stenosis, spondylolysis, and pseudo-articulations were not more common in patients with LSTV than those without (Elster, 1989). However, disc herniations were more likely to occur at the segment above LSTV (Fig. 3b). Avimadje et al., discovered that 52.7 % of patients with lumbar disc herniation also exhibited LSTV, versus only 18.3 % in the control group, suggesting a significant correlation and identifying LSTV as a risk factor for disc herniation (Avimadje et al., 1999). We attribute the discrepancy in conclusions to two key factors in Elster's experiment. First, the overly stringent criteria for diagnosing lesions excluded mild disc herniation caused by LSTV from the statistical analysis. Second, the study only included “referred patients with existing low back pain” and lacked a reference group of the general population. These two limitations collectively led Elster to draw conclusions that differ from those of recent studies.

Otani's 2001 study of 508 patients highlighted a higher incidence rate of conditions among those with LSTV, who also tended to be younger than their LSTV-free counterparts (Otani et al., 2001). Typically, the symptomatic disc level was located just above the LSTV. For unilateral LSTV cases, herniations above LSTV often appeared on the fused side, whereas herniations below LSTV were more common on the non-fused side. This is because the segment above the fused side, acting as four vertebrae, is under greater pressure and thus more prone to herniation. Conversely, decreased or absent mobility on the fused side below LSTV increases mobility on the non-fused side, leading to disc degeneration or herniation. Despite ongoing debates regarding LSTV's pain relationship, the literature consensus points to a higher prevalence of LSTV in degenerative spinal disease cases. Excessive movement and abnormal torque at the level above LSTV, especially on the fused side, coupled with restricted movement in the intervertebral space below LSTV, contribute to degenerative changes above LSTV. Vergauwen et al., in 1997, reported that disc herniations and/or extrusions were more frequent at levels adjacent to LSTV compared to non-LSTV patients (45.3 % vs. 30.3 %) (Vergauwen et al., 1997). Similar trends were observed for disc degeneration (52.8 % vs. 28 %), facet joint degeneration (60.4 % vs. 42.6 %), and neural foraminal stenosis (52.8 % vs. 27.9 %).

In a 2024 study conducted by Stylianos Kapetanakis, it was similarly observed that lumbar disc herniation in patients with LSTV primarily affected adjacent segments (adjacent segments: same segment = 72.1 %: 27.9 %), and the herniation direction was predominantly toward the contralateral side (contralateral side: ipsilateral side = 61.7 %: 38.3 %) (Kapetanakis et al., 2025).

In essence, LSTV's impact on adjacent discs may parallel the development mechanism of adjacent segment disease (ASD) following fusion surgery. Given L4/5's vulnerability to degeneration in LSTV contexts, Adolescent Idiopathic Scoliosis (AIS) patients should consider extending distal fusion to L3 instead of L4, preserving L3/4 mobility to slow L4/5's degenerative progression. Yamauchi et al., in 2023, noted a 24.5 % LSTV prevalence among Lenke 5C type AIS patients. For Lenke 5C type AIS patients with LSTV and LIV positioned at L3, the postoperative tilt at L4 was significantly more pronounced than in those without LSTV (Yamauchi et al., 2023). Thus, for AIS with significant lumbar curves and accompanying LSTV, distal fusion at L3, rather than L4, is advisable.

### 8.3 The surgical challenges posed by LSTV due to anatomical anomalies

Lumbosacral Transitional Vertebrae (LSTV) represent congenital anomalies at the L5-S1 segment, characterized by either the sacralization of the most distal lumbar vertebra or the lumbarization of the most proximal sacral vertebra (Fig. 3c). This deviation in anatomy significantly increases the risk of surgical mis-leveling (Mahato, 2013a; Chang & Nakagawa, 2004; Lian et al., 2018; Hou et al., 2018; Suzuki, 1987; Lisheng et al., 2023; Iwasaki et al., 2017; Huang et al., 2021; Takata et al., 2014; Li et al., 2014; Jenkins et al., 2023b; Santavirta et al., 1993; Paterson, 1893, 1894; Almeida et al., 2009; McCulloch & Waddell, 1980).

This issue stems from three main factors: 1. Inaccurate Intraoperative Localization: Focusing exclusively on localized lumbar MRI in the preoperative phase, without a holistic X-ray or full-spine MRI examination, often leads to reliance on intraoperative C-arm X-ray morphology correlated with preoperative MRI imagery for localization. However, the quality of intraoperative lateral images often falls short, and the conventionally identified last rectangular vertebra as L5, used for upward numbering, may actually be L4 or L6 in the presence of LSTV, leading to potential surgical mis-leveling. 2. Misidentification of Back Pain Sources: In 1993, Santavirta et al. performed surgeries on 16 symptomatic LSTV patients, with 13 still experiencing back pain and recurrences postoperatively (Santavirta et al., 1993). This underscores the need for cautious surgical selection. Surgery should be considered only for young patients without disc degeneration, where the pain is directly linked to LSTV. In patients with concurrent disc degeneration, accurately identifying the responsible segment is frequently fraught with errors. 3. Nerve Correspondence Variations due to Anatomical Variations in Bony Structures: In LSTV patients, the functionality of lumbar nerve roots may alter. In 1962, McCulloch et al. discovered through intraoperative observations that the innervation patterns of the lumbar nerve roots might change in the presence of a transitional vertebra at the lumbosacral region (McCulloch & Waddell, 1980). In cases of lumbarized LSTV, L5/L6 compression typically presents as L5 nerve dysfunction. It has been observed that symptoms arising from L6 nerve root compression bear similarity to those from L5 nerve root compression in a standard configuration, not S1, suggesting that in cases of S1 lumbarization, the S1 nerve root's function resembles more closely that of the L5 nerve root. This mismatch between the level of pathology and clinical symptoms amplifies the risk of operating at an incorrect segment.

### 8.4 Influence of LSTV on pedicle screw placement

The vertebral anatomy inherent to LSTV significantly affects the orientation required for inserting pedicle screws. In cases of sacral lumbarization (semi-circular shape), the lateral angle of pedicle screw insertion can be minimized (Ono et al., 2018). Conversely, with lumbar sacralization (triangular shape), a greater lateral angle is necessary to avoid penetrating the outer walls of the vertebral body (Fig. 3d). Occasionally, an intermediate form exists where the vertebra transitions from a semi-spherical shape above to a triangular shape below. Moreover, sacral lumbarization tends to reduce vertebral tilt, whereas lumbar sacralization increases it, which must be factored into screw placement strategies. Research indicates that asymmetry in the angles of pedicle roots in LSTV occurs in 9.29 % of cases, significantly higher than the 0.54 % observed in the general population (Mahato, 2011). This higher rate of pedicle root asymmetry also suggests lateral differences in nerve root anatomy, necessitating careful consideration during pedicle screw placement. Thus, thorough preoperative assessment of pedicle morphology is essential in patients with LSTV, particularly for surgeries involving pedicle screws. Such evaluations can minimize the risk of misplacement during pedicle screw insertion in the lumbosacral region. Notably, for Types IIIa and IV LSTV, disparities in pedicle root morphology are evident, underscoring the developmental linkage between transverse processes, articular processes, and pedicles.

### 8.5 Impact of LSTV on discectomy outcomes

LSTV has a discernible effect on the clinical outcomes regarding pain intensity and recurrence rates following discectomy procedures (Zileli et al., 2024; Huang et al., 2021; Shin et al., 2019; Ahn et al., 2017). As such, surgical interventions should proceed with caution, especially given the higher likelihood of persistent postoperative back pain in patients with LSTV. Shin et al.'s findings reveal a stark contrast in the occurrence of LSTV between patients with and without surgical recurrence-52.4 % in the recurrence group versus 7.1 % in the non-recurrence group (Shin et al., 2019). Multifactorial logistic regression analysis further identifies LSTV and an increased Sagittal Range of Motion (SROM) as significant predictors for the recurrence of disc herniation at the L4-L5 level. The heightened risk of re-herniation associated with LSTV may stem from biomechanical alterations in the lumbosacral area (Fig. 3e).

In their treatment of LSTV patients with lumbar disc degeneration using full-endoscopic surgery, Shen et al. observed that the mean VAS scores for low back pain and lower limb pain at post-operation and final follow-up decreased significantly compared with baseline values, and only 2.2 % of patients had poor prognosis regarding low back pain and lower limb pain (Shen et al., 2023).

In the study by Stylianos Kapetanakis, it was found that patients with LSTV and Bertolotti's Syndrome who underwent microdiscectomy exhibited favorable clinical outcomes, including functional recovery and pain relief, as well as a notable improvement in Health-Related Quality of Life during a 5-year follow-up period (Kapetanakis et al., 2025). These findings confirm the efficacy and safety of endoscopic surgery in the treatment of this disease.

However, the propensity for increased re-herniation in LSTV patients could prompt consideration for more aggressive surgical options, either in the initial discectomy or in planning for fusion as a primary or subsequent intervention. Nevertheless, Huang et al.'s research suggests that LSTV does not adversely impact the short-term clinical outcomes of adolescent Percutaneous Endoscopic Lumbar Discectomy (PELD) in terms of pain alleviation, functional recovery, or the necessity for subsequent surgeries (Huang et al., 2021).

### 8.6 The influence of LSTV on anterior surgical approaches

In cases involving LSTV, the atypical bony structures are often accompanied by variations in vascular anatomy, elevating the risk of vascular injuries (Becker et al., 2023; Moreau et al., 2016; Gong et al., 2023; Chung et al., 2021; Weiner et al., 2001). Notably, a prominent vein frequently situated anterior to LSTV necessitates meticulous preoperative examination of these anomalies in radiographs to significantly reduce the risk of severe vascular complications during surgeries utilizing an anterior approach. In patients with LSTV, the confluence of the iliac and inferior vena cava vessels typically overlays the intervertebral space, with the median sacral vessels joining the iliac at a position usually more posterior. The entry points of ascending lumbar veins, positioned closer to the iliac-inferior vena cava junction, form a substantial vascular coverage in front of the intervertebral space. Additionally, the median sacral and ascending lumbar vessels may exhibit enlargement (Fig. 3f), complicating access to this region due to the necessity for excessive vascular retraction, which could result in thrombosis, embolism, or potentially life-threatening tears and bleeding. Weiner and colleagues highlighted those alterations in the vascular anatomy of LSTV patients led to modifications in the surgical approach in 11 of 12 cases, with successful area access in only one instance (Weiner et al., 2001). For Anterior Lumbar Interbody Fusion (ALIF) procedures, it is imperative for both spine and access surgeons to preoperatively evaluate the aorta and inferior vena cava bifurcation. The presence of LSTV indicates a markedly reduced L5/S1 window, making the identification of smaller, yet significant, venous structures critical, particularly when LSTV is present. Similarly, in Oblique Lateral Interbody Fusion (OLIF) procedures targeting L4/5 fusion, damaging the ascending lumbar vessels can lead to disastrous outcomes.

## 9 Limitations

This study has several limitations. First and foremost, the literature currently lacks high-level evidence concerning the anatomy of the lumbosacral transitional area.Most of the studies included in this review are based on Level III or Level IV evidence. Additionally, as with all retrospective analyses, heterogeneity among the studies represents a limiting factor in this review. Specifically, variations in the standardized reporting of lumbosacral transitional vertebrae (LSTV) across different studies make it difficult to conduct a deeper investigation of the condition through meta-analysis, which undoubtedly stands out as a significant limitation of this study.

## 10 Conclusion

Considering the relatively common occurrence of LSTV within the population, it's imperative that considerations for surgical segment selection and parameter measurements include the presence of LSTV, emphasizing its clinical relevance to the development and prognosis of associated conditions. 1) There exists a definitive correlation between LSTV and LBP; 2) LSTV is associated with disc herniation and the alignment of the segment above it; 3) LSTV dictates the selection of distal fixation vertebrae in AIS; 4) LSTV complicates intraoperative surgical segment determination; 5) LSTV affects pedicle screw placement in the terminal vertebra; 6) LSTV has implications for the outcomes and recurrence rates post-discectomy; 7) LSTV challenges the execution of anterior surgical approaches. Future studies, with more extensive sample sizes and prolonged follow-up periods, are poised to further elucidate these clinical implications, thereby enhancing treatment guidance.

## Ethics approval and consent to participate

Not applicable.

## Consent for publication

Not applicable.

## Availability of data and materials

Not applicable.

## Authors' contributions

All authors contributed to the study conception and design. L.X, J.C.K, W.J and H.F analyzed the data. J.C.K,X.Z,C.M and C.Y interpreted the patient data. C.Y,W.J and Y.Z supervised the review. The first draft of the manuscript was written by H.F, J.C.K and L.X. And all authors commented on previous versions of the manuscript. All authors read and approved the final manuscript.

## Competing interests

The authors have no relevant financial or non-financial interests to disclose.
